# A jumping profile Hidden Markov Model and applications to recombination sites in HIV and HCV genomes

**DOI:** 10.1186/1471-2105-7-265

**Published:** 2006-05-22

**Authors:** Anne-Kathrin Schultz, Ming Zhang, Thomas Leitner, Carla Kuiken, Bette Korber, Burkhard Morgenstern, Mario Stanke

**Affiliations:** 1 Institute of Microbiology and Genetics, University of Göttingen, Goldschmidtstr. 1, 37077 Göttingen, Germany; 2 Theoretical Division, Los Alamos National Laboratory, Los Alamos, NM 87545, USA; 3 The Santa Fe Institute, Santa Fe, NM 87501, USA

## Abstract

**Background:**

Jumping alignments have recently been proposed as a strategy to search a given multiple sequence alignment *A *against a database. Instead of comparing a database sequence *S *to the multiple alignment or profile as a whole, *S *is compared and aligned to individual sequences from *A*. Within this alignment, *S *can *jump *between different sequences from *A*, so different parts of *S *can be aligned to different sequences from the input multiple alignment. This approach is particularly useful for dealing with *recombination *events.

**Results:**

We developed a *jumping profile Hidden Markov Model *(jpHMM), a probabilistic generalization of the jumping-alignment approach. Given a partition of the aligned input sequence family into known sequence *subtypes*, our model can jump between states corresponding to these different subtypes, depending on which subtype is locally most similar to a database sequence. *Jumps *between different subtypes are indicative of intersubtype recombinations. We applied our method to a large set of genome sequences from *human immunodeficiency virus *(HIV) and *hepatitis C virus *(HCV) as well as to simulated recombined genome sequences.

**Conclusion:**

Our results demonstrate that jumps in our jumping profile HMM often correspond to recombination breakpoints; our approach can therefore be used to detect recombinations in genomic sequences. The recombination breakpoints identified by jpHMM were found to be significantly more accurate than breakpoints defined by traditional methods based on comparing single representative sequences.

## Background

*Profile Hidden Markov Models *[1] are a popular way of modelling nucleic-acid or protein sequence families for database searching, see [2] for a review. Like other Hidden Markov Models (HMMs), profile HMMs consist of so-called *states *that can *emit *symbols of the underlying alphabet, i.e. nucleotides or amino acids [3]. *Transitions *are possible between these states, and a DNA or protein sequence is thought to be generated by a *path Q *through the model beginning with a special *begin *state and ending with an *end *state. There are probabilities (*a*) for possible transitions from one state to another and (*b*) for the emission of symbols at a given state. The states together with the possible transitions between them are called the *topology *of the model while the corresponding transition and emission probabilities are called its *parameters*. A sequence *S *is generated by the model with a certain probability *P*(*S*). In general, a sequence *S *can be generated by more than one path *Q *through the model. For a given sequence *S*, the well-known *Viterbi Algorithm *[4] finds the most probable path that generates *S*. More precisely, the algorithm finds a path Q* that maximizes the conditional probability *P*(*Q|S*) which is equivalent to maximizing the joint probability *P*(*Q, S*). For a general introduction to HMMs, see [5].

**Figure 1:**
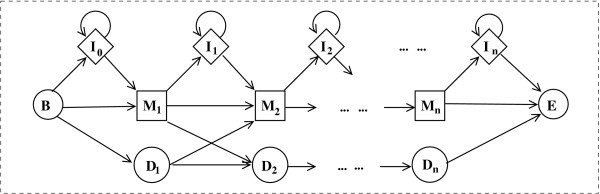
A profile hidden Markov model as introduced by Krogh et al. [1]. Squares indicate match states (*M*_*i*_), diamonds insert states (*I*_*i*_) and circles delete states (*D*_*i*_). Possible transitions are shown as arrows. *Begin *state (*B*), *End *state (*E*) and delete states (*D*_*i*_) are *silent *states, *i.e*. they do not emit symbols of the alphabet.

The starting point for a profile HMM is a multiple alignment of a sequence family. Columns of the alignment are modeled as states of the HMM. These states are called *match states *and are denoted by *M*_*i*_; the indexing is such that the alignment column associated with a match state *M*_*i *_is to the left of the column associated with *M*_*j *_whenever *i < j*. Emission probabilities for a match state *M*_*i *_depend on nucleotide or amino acid frequencies in the respective alignment column. In general, not every column of the input multiple alignment corresponds to a match state, but only those columns that have a certain minimum number of non-gap characters are modeled as match states. Columns that correspond to match states are called *consensus columns*. State transitions are possible from one match state *M*_*i *_to the next match state *M*_*i*+1_. To account for insertions and deletions, additional states are defined. *Insert states I*_*i *_can emit additional symbols while *delete states D*_*i *_can be used to omit one or more match states in the model. As the *Begin *and *End *states, delete states are *silent*, i.e. they do not emit any symbols. Figure 1 shows the topology of a profile HMM. An insert state *I*_*i *_is located between the match states *M*_*i *_and *M*_*i*+1 _and there are possible transitions from *M*_*i *_to *I*_*i *_and from *I*_*i *_to *M*_*i*+1 _such that an additional character can be inserted between *M*_*i *_and *M*_*i*+1_. By contrast, a deletion state *D*_*i *_is in the same column as a match state *M*_*i*_. There are possible transitions from *M*_*i-*1 _to *D*_*i *_and from *D*_*i *_to *M*_*i*+1 _to circumvent match state *M*_*i*_. Profile HMMs are frequently used tools for database searching. They are slower but more accurate than standard local-alignment approaches such as BLAST [6]. The best known implementation of profile HMMs is Sean Eddy's software program *HMMer *[2,7].

*Jumping alignments *have been proposed by Spang *et al*. as a new approach to database searching [8,9]. Like profile HMMs, jumping alignments start with a *multiple alignment A *of a sequence family, and database sequences *S *are compared to *A*. But unlike in standard methods, the database sequence is not aligned to the query multiple alignment *A *or to the corresponding profile as a whole, but the program aligns segments from the database sequence *S *to *single *sequences from the multiple alignment *A*. Each position of *S *is aligned with only one sequence from *A*, the so-called *reference sequence *for this position. Within one alignment, the program can *jump *between the reference sequences. For such jumps a *penalty *is imposed similar to the gap penalties that are used in standard alignment algorithms.

**Figure 2:**
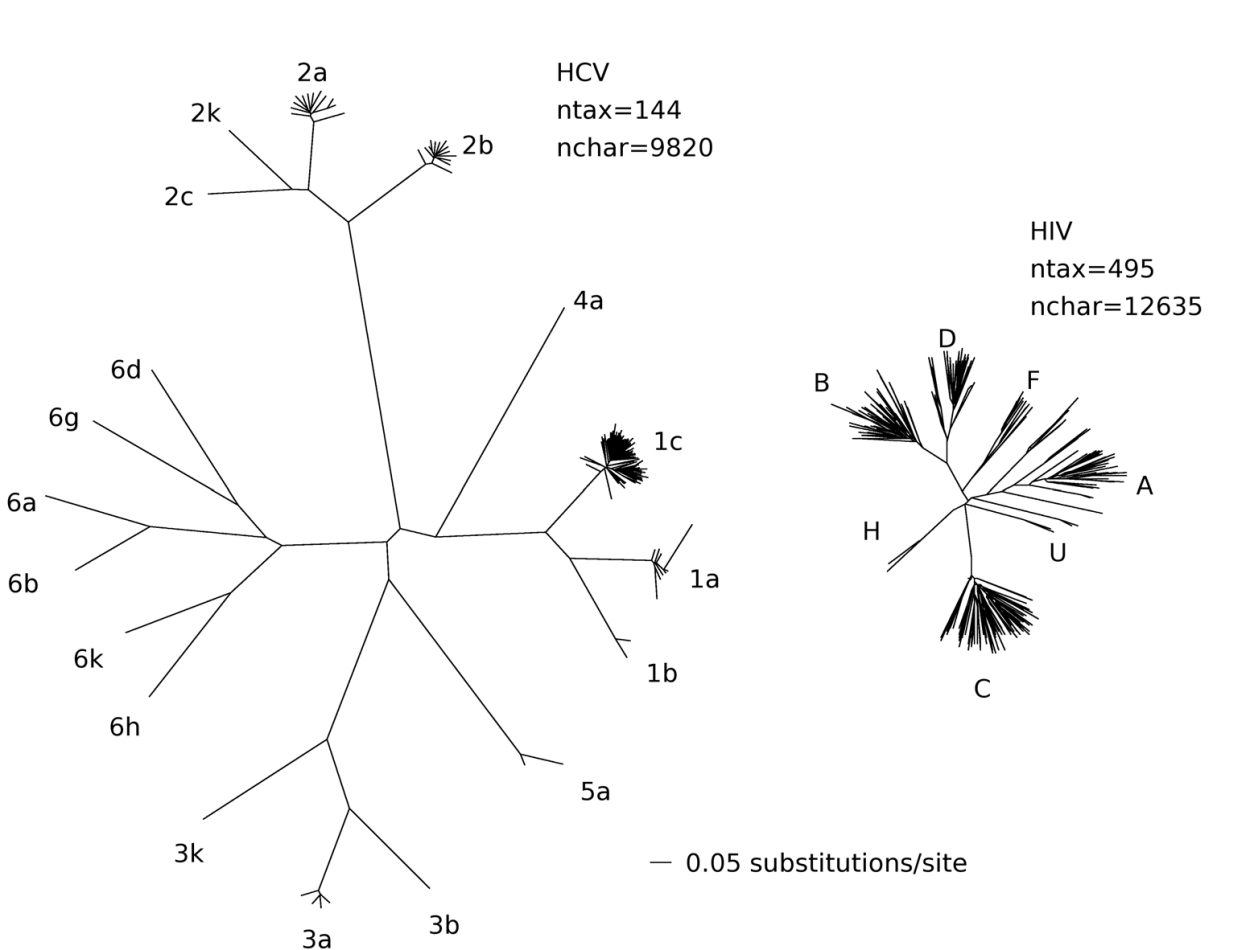
Complete genome trees of the hepatitis C and HIV-1 (M group) viruses. The trees are drawn on the same scale. Only non-recombinant complete genomes have been included. The trees are based on manually curated alignments containing only one sequence per patient. The optimalization method was maximum-likelihood, as implemented in the GAML/Garli program (Zwickl et al. 2005, in preparation).

In the present paper, we describe a novel approach to compare a single nucleic acid or protein sequence to a multiple alignment of a sequence family. Our approach combines the above outlined methods and can be seen as a probabilistic generalization of the jumping-alignment approach. We therefore call our method *jumping profile Hidden Markov Model (jpHMM)*. The proposed tool is a flexible method for database searching; it is particularly useful if sequence recombinations have to be taken into account. In the present study, we apply our method to localize phylogenetic breakpoints in viral genome sequences. We applied our approach to identify genomic recombinations in HIV and HCV and to classify subtypes. Accurate classification of HIV and HCV sequences is of crucial importance for epi-demiological monitoring as well as for the design of molecular detection systems and potential vaccines. HIV and HCV are among the most genetically variable organisms known. Based on phylogenetic clustering, these viruses have been classified into clades (Figure 2). The classification is not always trivial, because genetic forms that do not cluster within the phylogenetic clusters exist, and for both viruses recombinants have been discovered that make the classification more obscure. Furthermore, some genes and genome segments do not contain enough information to resolve the subtypes, especially when DNA sequences become too short.

Most classification methods depend on an accurate sequence alignment, and those that do not, still depend on pair-wise comparisons between a query sequence and some set of reference sequences. Since the subtypes are phylogenetically defined, tree building is the gold standard. Reconstructing accurate phylogenetic trees is, however, neither trivial nor easy to incorporate in automatic screening procedures. In recent years, many methods have been developed to detect genomic recombinations based on phylogenetic trees, sequence patterns and population genetics. In the virology field, the most popular methods have been based on pair-wise genetic distance calculations. Generally, HIV recombination is detected based on pairwise distances [10] and breakpoint locations are defined based on a method called "informative sites analysis" [11,12] prior to generating phylogenies, which are then used to validate the level of support for recombination in different genomic regions. Alternatively a *bootscan *is performed to determine whether phylogenetic branching patterns differ in trees constructed based on a sliding window approach [13]. Informative sites analysis divides the sequence at the midpoint between the substitutions that mark the dividing point that gives the most support for the recombination events using a chi square test; single representatives of the two recombinant clades are compared to the query sequence. In contrast, the jpHMM approach that we propose in this paper enables defining breakpoints based on a model derived from the full alignment of a particular clade; this is particularly useful when the precise parental strain is not known, rather a parental lineage is defined.

HIV-1 is classified into three major phylogenetic groups, called M, N and O, that arose due to separate introductions of SIVs from chimpanzees into humans [14]. The M group, which is responsible for the HIV pandemic, is further divided into ten subtypes, some of which have been even further subdivided into sub-subtypes [15]. Inter-subtype recombination is extremely common among HIV-1 subtypes [16]. Identifying intersubtype recombinants is important from many perspectives, giving insights into such issues as molecular epidemiology, viral evolution, and indirectly, the frequency of dual infections.

For hepatitis C, the picture is even more complex; there are currently 6 major genotypes, each subdivided into subtypes, of which there can be dozens. To curb the explosion in new subtypes, it was recently decided that new subtypes will only be named when there are at least three unrelated samples for them [17]. Recombinants that have been epidemiologically successful exist for both viruses, and are called *CRFs *in HIV and *RFs *in HCV, for *(circulating) recombinant forms*. HIV CRFs are common, and they often emerge as the dominant clade in a regional epidemic [18]. More precise breakpoint definitions will help in identifying and tracking HIV CRFs.

Currently only a small number of naturally occurring recombinants have been identified for the hepatitis C virus, despite frequent dual infection [19-24]. Until 2005, only one circulating recombinant form had been described, from St. Petersburg [25]. However, the discovery of new recombinants does appear to be speeding up, with two recent publications describing new circulating recombinants between genotypes 2a and 2c from Peru [26], and between genotypes 2i and 6h from Vietnam (S. Noppornpanth, unpublished results). It is very likely that more recombinants will be discovered in the near future. Discovery and accurate characterization of new recombinants is hampered by the scarcity of complete genome sequences for most of the less frequent HCV genotypes.

Recombinants found for hepatitis C so far are simple in structure, none of them appear to combine fragments from more than two genotypes and all appear to contain only one breakpoint. Thus, characterizing HCV recombinants found to date is a simpler task than characterizing the complex recombinants that are often found in HIV (for review, see [15]), for a specific example of the complexity, see [27]. However, given the improved sampling and sequencing capacity in HCV and the associated growing frequency of detection of recombinants, it will be increasingly important to have a tool that can reliably and efficiently identify recombinants and locate their breakpoints.

## Results

### Jumping profile Hidden Markov Model

A jumping profile Hidden Markov Model (jpHMM) as introduced in this section combines profile HMMs with the jumping alignment (JALI) approach introduced by Spang et al. [9]. The data that a jpHMM models are a sequence family S
 MathType@MTEF@5@5@+=feaafiart1ev1aaatCvAUfKttLearuWrP9MDH5MBPbIqV92AaeXatLxBI9gBamrtHrhAL1wy0L2yHvtyaeHbnfgDOvwBHrxAJfwnaebbnrfifHhDYfgasaacH8akY=wiFfYdH8Gipec8Eeeu0xXdbba9frFj0=OqFfea0dXdd9vqai=hGuQ8kuc9pgc9s8qqaq=dirpe0xb9q8qiLsFr0=vr0=vr0dc8meaabaqaciaacaGaaeqabaWaaeGaeaaakeaaimaacqWFse=uaaa@3845@= {*S*_1_,...,*S*_*n*_} together with a multiple sequence alignment *A *of S
 MathType@MTEF@5@5@+=feaafiart1ev1aaatCvAUfKttLearuWrP9MDH5MBPbIqV92AaeXatLxBI9gBamrtHrhAL1wy0L2yHvtyaeHbnfgDOvwBHrxAJfwnaebbnrfifHhDYfgasaacH8akY=wiFfYdH8Gipec8Eeeu0xXdbba9frFj0=OqFfea0dXdd9vqai=hGuQ8kuc9pgc9s8qqaq=dirpe0xb9q8qiLsFr0=vr0=vr0dc8meaabaqaciaacaGaaeqabaWaaeGaeaaakeaaimaacqWFse=uaaa@3845@. In addition, we assume that we have a partition of S
 MathType@MTEF@5@5@+=feaafiart1ev1aaatCvAUfKttLearuWrP9MDH5MBPbIqV92AaeXatLxBI9gBamrtHrhAL1wy0L2yHvtyaeHbnfgDOvwBHrxAJfwnaebbnrfifHhDYfgasaacH8akY=wiFfYdH8Gipec8Eeeu0xXdbba9frFj0=OqFfea0dXdd9vqai=hGuQ8kuc9pgc9s8qqaq=dirpe0xb9q8qiLsFr0=vr0=vr0dc8meaabaqaciaacaGaaeqabaWaaeGaeaaakeaaimaacqWFse=uaaa@3845@ into *k subtypes *S
 MathType@MTEF@5@5@+=feaafiart1ev1aaatCvAUfKttLearuWrP9MDH5MBPbIqV92AaeXatLxBI9gBamrtHrhAL1wy0L2yHvtyaeHbnfgDOvwBHrxAJfwnaebbnrfifHhDYfgasaacH8akY=wiFfYdH8Gipec8Eeeu0xXdbba9frFj0=OqFfea0dXdd9vqai=hGuQ8kuc9pgc9s8qqaq=dirpe0xb9q8qiLsFr0=vr0=vr0dc8meaabaqaciaacaGaaeqabaWaaeGaeaaakeaaimaacqWFse=uaaa@3845@_1_,...,S
 MathType@MTEF@5@5@+=feaafiart1ev1aaatCvAUfKttLearuWrP9MDH5MBPbIqV92AaeXatLxBI9gBamrtHrhAL1wy0L2yHvtyaeHbnfgDOvwBHrxAJfwnaebbnrfifHhDYfgasaacH8akY=wiFfYdH8Gipec8Eeeu0xXdbba9frFj0=OqFfea0dXdd9vqai=hGuQ8kuc9pgc9s8qqaq=dirpe0xb9q8qiLsFr0=vr0=vr0dc8meaabaqaciaacaGaaeqabaWaaeGaeaaakeaaimaacqWFse=uaaa@3845@_*k *_such that each sequence *S*_*i *_belongs to exactly one of the subtypes. Our jpHMM approach can be seen as a generalization of the 'jumping alignment' algorithm. In JALI, each position of a database sequence is aligned to one reference sequence *S*_*i *_∈ S
 MathType@MTEF@5@5@+=feaafiart1ev1aaatCvAUfKttLearuWrP9MDH5MBPbIqV92AaeXatLxBI9gBamrtHrhAL1wy0L2yHvtyaeHbnfgDOvwBHrxAJfwnaebbnrfifHhDYfgasaacH8akY=wiFfYdH8Gipec8Eeeu0xXdbba9frFj0=OqFfea0dXdd9vqai=hGuQ8kuc9pgc9s8qqaq=dirpe0xb9q8qiLsFr0=vr0=vr0dc8meaabaqaciaacaGaaeqabaWaaeGaeaaakeaaimaacqWFse=uaaa@3845@. By contrast, jpHMM aligns parts of the input sequence to entire *subtypes *of the input multiple alignment. Thus, JALI corresponds to the special case in our approach where each subtype S
 MathType@MTEF@5@5@+=feaafiart1ev1aaatCvAUfKttLearuWrP9MDH5MBPbIqV92AaeXatLxBI9gBamrtHrhAL1wy0L2yHvtyaeHbnfgDOvwBHrxAJfwnaebbnrfifHhDYfgasaacH8akY=wiFfYdH8Gipec8Eeeu0xXdbba9frFj0=OqFfea0dXdd9vqai=hGuQ8kuc9pgc9s8qqaq=dirpe0xb9q8qiLsFr0=vr0=vr0dc8meaabaqaciaacaGaaeqabaWaaeGaeaaakeaaimaacqWFse=uaaa@3845@_*i *_consists of exactly one sequence. As with standard profile HMMs, each match state in our model is derived from one column of the input alignment *A*. However, in our model we define match states specific for the subtypes. Thus, a column in the query alignment *A *may correspond to up to *k *match states, and a match state is specified by two indices, the corresponding column of the multiple alignment *A *and the subtype it belongs to.

For a given subtype S
 MathType@MTEF@5@5@+=feaafiart1ev1aaatCvAUfKttLearuWrP9MDH5MBPbIqV92AaeXatLxBI9gBamrtHrhAL1wy0L2yHvtyaeHbnfgDOvwBHrxAJfwnaebbnrfifHhDYfgasaacH8akY=wiFfYdH8Gipec8Eeeu0xXdbba9frFj0=OqFfea0dXdd9vqai=hGuQ8kuc9pgc9s8qqaq=dirpe0xb9q8qiLsFr0=vr0=vr0dc8meaabaqaciaacaGaaeqabaWaaeGaeaaakeaaimaacqWFse=uaaa@3845@_*i*_, a column is modeled as a match state only if it is a consensus column for that subtype. Consequently, a column *i *in the alignment *A *may be modeled as a match state for some of the subtypes but not for other subtypes. In addition to the match states, we have distinct insert and delete states for each subtype just as in standard profile HMMs. In our notation, match state *M*_*i,j *_is the *j*-th match state within the *i*-th subtype, and *I*_*i,j *_and *D*_*i,j *_are the insert and delete states corresponding to *M*_*i,j*_. There is a single *Begin *state and a single *End *state, respectively, for the entire model. Further, there are general, not subtype-specific insert and delete states just after the *Begin *state and just before the *End *state. From the delete state immediately after the *Begin *state, each match state from each subtype can be reached. Similarly, from each match state, the delete state directly before the *End *state can be reached. These states have been introduced to deal with the fact that the sequences are often incompletely sequenced and are missing the initial or terminal part.

**Figure 3:**
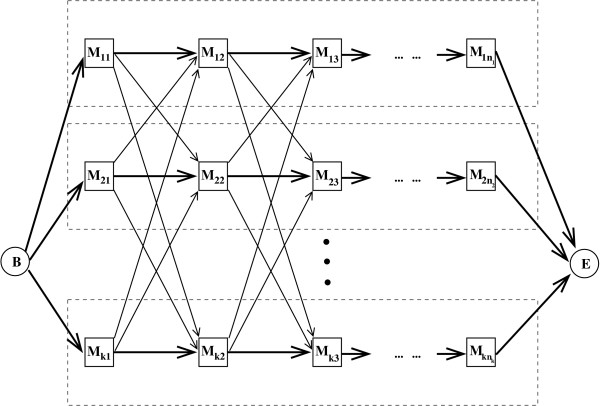
Simplified topology of a jumping profile HMM (jpHMM). The sequence family S
 MathType@MTEF@5@5@+=feaafiart1ev1aaatCvAUfKttLearuWrP9MDH5MBPbIqV92AaeXatLxBI9gBamrtHrhAL1wy0L2yHvtyaeHbnfgDOvwBHrxAJfwnaebbnrfifHhDYfgasaacH8akY=wiFfYdH8Gipec8Eeeu0xXdbba9frFj0=OqFfea0dXdd9vqai=hGuQ8kuc9pgc9s8qqaq=dirpe0xb9q8qiLsFr0=vr0=vr0dc8meaabaqaciaacaGaaeqabaWaaeGaeaaakeaaimaacqWFse=uaaa@3845@ is partitioned into *k *subtypes S
 MathType@MTEF@5@5@+=feaafiart1ev1aaatCvAUfKttLearuWrP9MDH5MBPbIqV92AaeXatLxBI9gBamrtHrhAL1wy0L2yHvtyaeHbnfgDOvwBHrxAJfwnaebbnrfifHhDYfgasaacH8akY=wiFfYdH8Gipec8Eeeu0xXdbba9frFj0=OqFfea0dXdd9vqai=hGuQ8kuc9pgc9s8qqaq=dirpe0xb9q8qiLsFr0=vr0=vr0dc8meaabaqaciaacaGaaeqabaWaaeGaeaaakeaaimaacqWFse=uaaa@3845@_1_,...,S
 MathType@MTEF@5@5@+=feaafiart1ev1aaatCvAUfKttLearuWrP9MDH5MBPbIqV92AaeXatLxBI9gBamrtHrhAL1wy0L2yHvtyaeHbnfgDOvwBHrxAJfwnaebbnrfifHhDYfgasaacH8akY=wiFfYdH8Gipec8Eeeu0xXdbba9frFj0=OqFfea0dXdd9vqai=hGuQ8kuc9pgc9s8qqaq=dirpe0xb9q8qiLsFr0=vr0=vr0dc8meaabaqaciaacaGaaeqabaWaaeGaeaaakeaaimaacqWFse=uaaa@3845@_*k*_. Each subtype is modeled by a profile HMM here pictured as a dashed box. Arrows indicate possible transitions between states within the same subtypes and transitions between different subtypes, so-called *jumps*. For clarity we omit insert and delete states of the profile HMMs and sketch a case where the first three columns are consensus columns.

**Figure 4:**
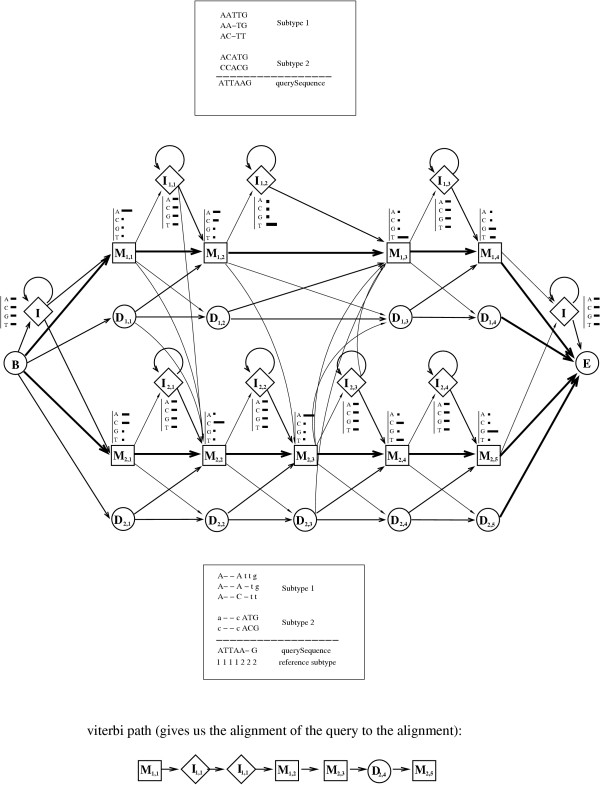
A toy example of a jumping profile Hidden Markov Model. This is build from a multiple sequence alignment of nucleotides with two subtypes. The first subtype consists of three sequences with four consensus columns, the second subtype consists of two sequences with five consensus columns. With each match and insert state a vector is associated for the emission probability values corresponding to the nucleotides A, C, G and T. For clarity, some transitions are omitted. Also, the figure does not show the delete states immediately after *B *from which each match state can be reached nor the delete state immediately before *E *that can be reached from each match state. Fat lines indicate high transition probabilities, thin lines correspond to low probabilities.

Note that the sub-model associated with a subtype S
 MathType@MTEF@5@5@+=feaafiart1ev1aaatCvAUfKttLearuWrP9MDH5MBPbIqV92AaeXatLxBI9gBamrtHrhAL1wy0L2yHvtyaeHbnfgDOvwBHrxAJfwnaebbnrfifHhDYfgasaacH8akY=wiFfYdH8Gipec8Eeeu0xXdbba9frFj0=OqFfea0dXdd9vqai=hGuQ8kuc9pgc9s8qqaq=dirpe0xb9q8qiLsFr0=vr0=vr0dc8meaabaqaciaacaGaaeqabaWaaeGaeaaakeaaimaacqWFse=uaaa@3845@_*i *_in our jpHMM corresponds to a standard profile HMM for S
 MathType@MTEF@5@5@+=feaafiart1ev1aaatCvAUfKttLearuWrP9MDH5MBPbIqV92AaeXatLxBI9gBamrtHrhAL1wy0L2yHvtyaeHbnfgDOvwBHrxAJfwnaebbnrfifHhDYfgasaacH8akY=wiFfYdH8Gipec8Eeeu0xXdbba9frFj0=OqFfea0dXdd9vqai=hGuQ8kuc9pgc9s8qqaq=dirpe0xb9q8qiLsFr0=vr0=vr0dc8meaabaqaciaacaGaaeqabaWaaeGaeaaakeaaimaacqWFse=uaaa@3845@_*i*_. Thus, our model can be seen as the union of *k *standard profile HMMs with additional transitions between these standard HMMs. The underlying multiple alignment *A *induces a *quasi partial order relation *on the set of all states of our jpHMM. We say that a match or delete state *T *is (strictly) to the left of a match or delete state *R *if the alignment column associated to *T *is (strictly) to the left of the column associated with *R*. This ordering is related to the quasi partial order relation ∘*A *defined on the set of all *sites *of a multiple alignment introduced in [28] in the context of *consistency *of alignments. As for standard HMMs, the states of a jpHMM are connected by transitions to which transition probabilities are assigned. Transitions are possible *within *one subtype S
 MathType@MTEF@5@5@+=feaafiart1ev1aaatCvAUfKttLearuWrP9MDH5MBPbIqV92AaeXatLxBI9gBamrtHrhAL1wy0L2yHvtyaeHbnfgDOvwBHrxAJfwnaebbnrfifHhDYfgasaacH8akY=wiFfYdH8Gipec8Eeeu0xXdbba9frFj0=OqFfea0dXdd9vqai=hGuQ8kuc9pgc9s8qqaq=dirpe0xb9q8qiLsFr0=vr0=vr0dc8meaabaqaciaacaGaaeqabaWaaeGaeaaakeaaimaacqWFse=uaaa@3845@_*i *_as in standard profile HMMs, e.g. from one match state a transition is possible to the next match state or to the corresponding insert and delete states of S
 MathType@MTEF@5@5@+=feaafiart1ev1aaatCvAUfKttLearuWrP9MDH5MBPbIqV92AaeXatLxBI9gBamrtHrhAL1wy0L2yHvtyaeHbnfgDOvwBHrxAJfwnaebbnrfifHhDYfgasaacH8akY=wiFfYdH8Gipec8Eeeu0xXdbba9frFj0=OqFfea0dXdd9vqai=hGuQ8kuc9pgc9s8qqaq=dirpe0xb9q8qiLsFr0=vr0=vr0dc8meaabaqaciaacaGaaeqabaWaaeGaeaaakeaaimaacqWFse=uaaa@3845@_*i*_. In addition, our model allows transitions *between *different subtypes as shown in Figures 3 and 4. Transitions between subtypes are called *jumps*.

Transitions *between *subtypes are more complicated than *within *subtypes since not every alignment column is represented in every subtype as a match state. Thus, it is not obvious from which state in one subtype we can jump to which state in another subtype, so we need to specify which jumps between subtypes are allowed. Generally, there are two reasons to limit the number of possible jumps between states. (*a*) To reduce the computer resources required by our algorithm, we need to limit the number of possible transitions between states. (*b*) More importantly, we need to make sure that a path through our model cannot jump to the left or too far to the right. A jump to the left would have the biological meaning of a tandem repeat of a certain part of the sequence, which we do not allow. A jump to the right that overjumps consensus columns in one of the two subtypes involved in the jump means that some part of one of those subtypes is deleted with respect to the query sequence. This is possible but should be punished as in a standard profile HMM by using the alternative path, a chain of delete states. This exclusion of forward jumps similarly reduces the number of transitions as done in [29]. In our approach, we imposed the following rules:

r1 For two subtypes S
 MathType@MTEF@5@5@+=feaafiart1ev1aaatCvAUfKttLearuWrP9MDH5MBPbIqV92AaeXatLxBI9gBamrtHrhAL1wy0L2yHvtyaeHbnfgDOvwBHrxAJfwnaebbnrfifHhDYfgasaacH8akY=wiFfYdH8Gipec8Eeeu0xXdbba9frFj0=OqFfea0dXdd9vqai=hGuQ8kuc9pgc9s8qqaq=dirpe0xb9q8qiLsFr0=vr0=vr0dc8meaabaqaciaacaGaaeqabaWaaeGaeaaakeaaimaacqWFse=uaaa@3845@_*i *_and S
 MathType@MTEF@5@5@+=feaafiart1ev1aaatCvAUfKttLearuWrP9MDH5MBPbIqV92AaeXatLxBI9gBamrtHrhAL1wy0L2yHvtyaeHbnfgDOvwBHrxAJfwnaebbnrfifHhDYfgasaacH8akY=wiFfYdH8Gipec8Eeeu0xXdbba9frFj0=OqFfea0dXdd9vqai=hGuQ8kuc9pgc9s8qqaq=dirpe0xb9q8qiLsFr0=vr0=vr0dc8meaabaqaciaacaGaaeqabaWaaeGaeaaakeaaimaacqWFse=uaaa@3845@_*j*_, the algorithm can jump from a match state of S
 MathType@MTEF@5@5@+=feaafiart1ev1aaatCvAUfKttLearuWrP9MDH5MBPbIqV92AaeXatLxBI9gBamrtHrhAL1wy0L2yHvtyaeHbnfgDOvwBHrxAJfwnaebbnrfifHhDYfgasaacH8akY=wiFfYdH8Gipec8Eeeu0xXdbba9frFj0=OqFfea0dXdd9vqai=hGuQ8kuc9pgc9s8qqaq=dirpe0xb9q8qiLsFr0=vr0=vr0dc8meaabaqaciaacaGaaeqabaWaaeGaeaaakeaaimaacqWFse=uaaa@3845@_*i *_only to a match state or a delete state of S
 MathType@MTEF@5@5@+=feaafiart1ev1aaatCvAUfKttLearuWrP9MDH5MBPbIqV92AaeXatLxBI9gBamrtHrhAL1wy0L2yHvtyaeHbnfgDOvwBHrxAJfwnaebbnrfifHhDYfgasaacH8akY=wiFfYdH8Gipec8Eeeu0xXdbba9frFj0=OqFfea0dXdd9vqai=hGuQ8kuc9pgc9s8qqaq=dirpe0xb9q8qiLsFr0=vr0=vr0dc8meaabaqaciaacaGaaeqabaWaaeGaeaaakeaaimaacqWFse=uaaa@3845@_*j*_, and from an insert state or delete state of S
 MathType@MTEF@5@5@+=feaafiart1ev1aaatCvAUfKttLearuWrP9MDH5MBPbIqV92AaeXatLxBI9gBamrtHrhAL1wy0L2yHvtyaeHbnfgDOvwBHrxAJfwnaebbnrfifHhDYfgasaacH8akY=wiFfYdH8Gipec8Eeeu0xXdbba9frFj0=OqFfea0dXdd9vqai=hGuQ8kuc9pgc9s8qqaq=dirpe0xb9q8qiLsFr0=vr0=vr0dc8meaabaqaciaacaGaaeqabaWaaeGaeaaakeaaimaacqWFse=uaaa@3845@_*i *_a jump is possible only to a match state of S
 MathType@MTEF@5@5@+=feaafiart1ev1aaatCvAUfKttLearuWrP9MDH5MBPbIqV92AaeXatLxBI9gBamrtHrhAL1wy0L2yHvtyaeHbnfgDOvwBHrxAJfwnaebbnrfifHhDYfgasaacH8akY=wiFfYdH8Gipec8Eeeu0xXdbba9frFj0=OqFfea0dXdd9vqai=hGuQ8kuc9pgc9s8qqaq=dirpe0xb9q8qiLsFr0=vr0=vr0dc8meaabaqaciaacaGaaeqabaWaaeGaeaaakeaaimaacqWFse=uaaa@3845@_*j*_.

r2 A jump from a state *T *in S
 MathType@MTEF@5@5@+=feaafiart1ev1aaatCvAUfKttLearuWrP9MDH5MBPbIqV92AaeXatLxBI9gBamrtHrhAL1wy0L2yHvtyaeHbnfgDOvwBHrxAJfwnaebbnrfifHhDYfgasaacH8akY=wiFfYdH8Gipec8Eeeu0xXdbba9frFj0=OqFfea0dXdd9vqai=hGuQ8kuc9pgc9s8qqaq=dirpe0xb9q8qiLsFr0=vr0=vr0dc8meaabaqaciaacaGaaeqabaWaaeGaeaaakeaaimaacqWFse=uaaa@3845@_*i *_is possible only to the *left-most *state in S
 MathType@MTEF@5@5@+=feaafiart1ev1aaatCvAUfKttLearuWrP9MDH5MBPbIqV92AaeXatLxBI9gBamrtHrhAL1wy0L2yHvtyaeHbnfgDOvwBHrxAJfwnaebbnrfifHhDYfgasaacH8akY=wiFfYdH8Gipec8Eeeu0xXdbba9frFj0=OqFfea0dXdd9vqai=hGuQ8kuc9pgc9s8qqaq=dirpe0xb9q8qiLsFr0=vr0=vr0dc8meaabaqaciaacaGaaeqabaWaaeGaeaaakeaaimaacqWFse=uaaa@3845@_*j *_that is *strictly to the right *of *T*.

r3 A jump from a state *M*_*i,k*_, *D*_*i,k *_or *I*_*i,k *_to a state in S
 MathType@MTEF@5@5@+=feaafiart1ev1aaatCvAUfKttLearuWrP9MDH5MBPbIqV92AaeXatLxBI9gBamrtHrhAL1wy0L2yHvtyaeHbnfgDOvwBHrxAJfwnaebbnrfifHhDYfgasaacH8akY=wiFfYdH8Gipec8Eeeu0xXdbba9frFj0=OqFfea0dXdd9vqai=hGuQ8kuc9pgc9s8qqaq=dirpe0xb9q8qiLsFr0=vr0=vr0dc8meaabaqaciaacaGaaeqabaWaaeGaeaaakeaaimaacqWFse=uaaa@3845@_*j *_that is to the right of *M*_*i,k*+1 _is not possible.

Rule r1 reduces the number of possible transitions in our model. Rules r2 and r3 ensure that there are no insertions or deletions introduced during a jump without using insert or delete states.

#### Parameter estimation

A jpHMM has a large number of parameters that need to be specified, namely the emission probabilities of match and insert states, the transition probabilities *within *subtypes and the probability of the jumps *between *different subtypes. With the exception of the probabilities of jumps, which is discussed below, the above probabilities can be estimated based on observed frequencies. Given the topology of the jpHMM, each of the sequences in the given multiple sequence alignment defines a unique path through the states, and gives rise to observed emissions and transitions. For example, a particular residue that is aligned in a consensus column is emitted from the corresponding match state of the subtype the respective sequence belongs to. To give another example, an insert region of length *l *gives rise to one transition from the preceding match state to the corresponding insert state, *l *- 1 transitions from that insert state to itself and one transition from the insert state to the next match state.

The generalized problem for estimating each emission distribution and each distribution of possible transitions out of a state is the following. We are given a count vector n→
 MathType@MTEF@5@5@+=feaafiart1ev1aaatCvAUfKttLearuWrP9MDH5MBPbIqV92AaeXatLxBI9gBaebbnrfifHhDYfgasaacH8akY=wiFfYdH8Gipec8Eeeu0xXdbba9frFj0=OqFfea0dXdd9vqai=hGuQ8kuc9pgc9s8qqaq=dirpe0xb9q8qiLsFr0=vr0=vr0dc8meaabaqaciaacaGaaeqabaqabeGadaaakeaacuWGUbGBgaWcaaaa@2E23@ = (*n*_1_,...,*n*_*s*_), where *s *is the number of emissions (or transitions, respectively) out of this state. For example, we have *s *= 4 in case of nucleotide emissions. *n*_*i *_is the number of times the *i*th emission (or transition) is observed. These observed frequencies n→
 MathType@MTEF@5@5@+=feaafiart1ev1aaatCvAUfKttLearuWrP9MDH5MBPbIqV92AaeXatLxBI9gBaebbnrfifHhDYfgasaacH8akY=wiFfYdH8Gipec8Eeeu0xXdbba9frFj0=OqFfea0dXdd9vqai=hGuQ8kuc9pgc9s8qqaq=dirpe0xb9q8qiLsFr0=vr0=vr0dc8meaabaqaciaacaGaaeqabaqabeGadaaakeaacuWGUbGBgaWcaaaa@2E23@ are distributed according to a multinomial distribution with parameters p→
 MathType@MTEF@5@5@+=feaafiart1ev1aaatCvAUfKttLearuWrP9MDH5MBPbIqV92AaeXatLxBI9gBaebbnrfifHhDYfgasaacH8akY=wiFfYdH8Gipec8Eeeu0xXdbba9frFj0=OqFfea0dXdd9vqai=hGuQ8kuc9pgc9s8qqaq=dirpe0xb9q8qiLsFr0=vr0=vr0dc8meaabaqaciaacaGaaeqabaqabeGadaaakeaacuWGWbaCgaWcaaaa@2E27@ = (*p*_1_,...,*p*_*s*_), where *p*_*i *_is the probability of observing option *i*. For this problem of estimating p→
 MathType@MTEF@5@5@+=feaafiart1ev1aaatCvAUfKttLearuWrP9MDH5MBPbIqV92AaeXatLxBI9gBaebbnrfifHhDYfgasaacH8akY=wiFfYdH8Gipec8Eeeu0xXdbba9frFj0=OqFfea0dXdd9vqai=hGuQ8kuc9pgc9s8qqaq=dirpe0xb9q8qiLsFr0=vr0=vr0dc8meaabaqaciaacaGaaeqabaqabeGadaaakeaacuWGWbaCgaWcaaaa@2E27@ given n→
 MathType@MTEF@5@5@+=feaafiart1ev1aaatCvAUfKttLearuWrP9MDH5MBPbIqV92AaeXatLxBI9gBaebbnrfifHhDYfgasaacH8akY=wiFfYdH8Gipec8Eeeu0xXdbba9frFj0=OqFfea0dXdd9vqai=hGuQ8kuc9pgc9s8qqaq=dirpe0xb9q8qiLsFr0=vr0=vr0dc8meaabaqaciaacaGaaeqabaqabeGadaaakeaacuWGUbGBgaWcaaaa@2E23@, we chose a Bayesian approach as in [30]. This means we assume a prior distribution on the set of all possible p→
 MathType@MTEF@5@5@+=feaafiart1ev1aaatCvAUfKttLearuWrP9MDH5MBPbIqV92AaeXatLxBI9gBaebbnrfifHhDYfgasaacH8akY=wiFfYdH8Gipec8Eeeu0xXdbba9frFj0=OqFfea0dXdd9vqai=hGuQ8kuc9pgc9s8qqaq=dirpe0xb9q8qiLsFr0=vr0=vr0dc8meaabaqaciaacaGaaeqabaqabeGadaaakeaacuWGWbaCgaWcaaaa@2E27@, and then estimate p→
 MathType@MTEF@5@5@+=feaafiart1ev1aaatCvAUfKttLearuWrP9MDH5MBPbIqV92AaeXatLxBI9gBaebbnrfifHhDYfgasaacH8akY=wiFfYdH8Gipec8Eeeu0xXdbba9frFj0=OqFfea0dXdd9vqai=hGuQ8kuc9pgc9s8qqaq=dirpe0xb9q8qiLsFr0=vr0=vr0dc8meaabaqaciaacaGaaeqabaqabeGadaaakeaacuWGWbaCgaWcaaaa@2E27@ by the following conditional expectation

E(p→
 MathType@MTEF@5@5@+=feaafiart1ev1aaatCvAUfKttLearuWrP9MDH5MBPbIqV92AaeXatLxBI9gBaebbnrfifHhDYfgasaacH8akY=wiFfYdH8Gipec8Eeeu0xXdbba9frFj0=OqFfea0dXdd9vqai=hGuQ8kuc9pgc9s8qqaq=dirpe0xb9q8qiLsFr0=vr0=vr0dc8meaabaqaciaacaGaaeqabaqabeGadaaakeaacuWGWbaCgaWcaaaa@2E27@|n→
 MathType@MTEF@5@5@+=feaafiart1ev1aaatCvAUfKttLearuWrP9MDH5MBPbIqV92AaeXatLxBI9gBaebbnrfifHhDYfgasaacH8akY=wiFfYdH8Gipec8Eeeu0xXdbba9frFj0=OqFfea0dXdd9vqai=hGuQ8kuc9pgc9s8qqaq=dirpe0xb9q8qiLsFr0=vr0=vr0dc8meaabaqaciaacaGaaeqabaqabeGadaaakeaacuWGUbGBgaWcaaaa@2E23@).

We model this prior knowledge using a Dirichlet distribution [30] which has parameters α→
 MathType@MTEF@5@5@+=feaafiart1ev1aaatCvAUfKttLearuWrP9MDH5MBPbIqV92AaeXatLxBI9gBaebbnrfifHhDYfgasaacH8akY=wiFfYdH8Gipec8Eeeu0xXdbba9frFj0=OqFfea0dXdd9vqai=hGuQ8kuc9pgc9s8qqaq=dirpe0xb9q8qiLsFr0=vr0=vr0dc8meaabaqaciaacaGaaeqabaqabeGadaaakeaaiiGacuWFXoqygaWcaaaa@2E64@ = (*α*_1_,...,*α*_*s*_). These parameters can be interpreted as pseudocounts that are added to the observed counts. For the emission probabilities we estimated the parameters α→
 MathType@MTEF@5@5@+=feaafiart1ev1aaatCvAUfKttLearuWrP9MDH5MBPbIqV92AaeXatLxBI9gBaebbnrfifHhDYfgasaacH8akY=wiFfYdH8Gipec8Eeeu0xXdbba9frFj0=OqFfea0dXdd9vqai=hGuQ8kuc9pgc9s8qqaq=dirpe0xb9q8qiLsFr0=vr0=vr0dc8meaabaqaciaacaGaaeqabaqabeGadaaakeaaiiGacuWFXoqygaWcaaaa@2E64@ of the prior distribution with a Maximum Likelihood approach [30] based on the input multiple alignment. For the transition probabilities we used the parameters α→
 MathType@MTEF@5@5@+=feaafiart1ev1aaatCvAUfKttLearuWrP9MDH5MBPbIqV92AaeXatLxBI9gBaebbnrfifHhDYfgasaacH8akY=wiFfYdH8Gipec8Eeeu0xXdbba9frFj0=OqFfea0dXdd9vqai=hGuQ8kuc9pgc9s8qqaq=dirpe0xb9q8qiLsFr0=vr0=vr0dc8meaabaqaciaacaGaaeqabaqabeGadaaakeaaiiGacuWFXoqygaWcaaaa@2E64@ of the prior distribution taken from [31]. Those were shown to perform better than the parameters derived by Maximum Likelihood.

In contrast to the transitions *within *a subtype of the jpHMM, jumps *between *subtypes cannot be observed in the input alignment data. Since we cannot estimate the corresponding jump probabilities from observed frequencies, we use a fixed, empirically derived value *P*_*j *_for the probability of observing a jump. If in a given state of the jpHMM, there are several possibilities for a jump, this probability is evenly distributed between the possible jumps. In other words, if we have *K *options to jump into another subtype, the probability of each of these jumps is given by *P*_*j*_/*K*. In the application of our program to the identification of HIV and HCV recombinants we use a jump probability of *P*_*j *_= 10^-9 ^which we derived by optimizing the results by comparing them to published HIV intersubtype recombination breakpoints. Taking into account that the transition and jump probabilities out of each state must sum up to 1, we scale the non-jump transition probabilities, i.e. the probabilities for transitions within the same subtype by multiplying them by (1 - *P*_*j*_), if jumps are possible out of this state.

#### Alignment algorithm and efficiency

The jumping alignment of a query sequence *S *= *s*_1_, *s*_2_,...,*s*_*n *_and a given multiple alignment is determined by searching the most probable path Q* through the jpHMM that emits *S *as described above. This is done with a dynamic programming algorithm, the Viterbi algorithm. For each position *i *= 1,..., *n *of the query sequence *S *and for each state *q *of the jpHMM we calculate the probability *δ*_*i*_(*q*) of the prefix *s*_1_,...,*s*_*i *_of the query sequence and the most probable path through the jpHMM ending in state *q *and emitting *s*_1_,...,*s*_*i*_. These probabilities are called Viterbi values and the following recursion holds.

δi+1(q)={max⁡q′{δi+1(q′)tq′q},if q is a delete state;max⁡q′{δi(q′)tq′q}eq,si+1,otherwise.
 MathType@MTEF@5@5@+=feaafiart1ev1aaatCvAUfKttLearuWrP9MDH5MBPbIqV92AaeXatLxBI9gBaebbnrfifHhDYfgasaacH8akY=wiFfYdH8Gipec8Eeeu0xXdbba9frFj0=OqFfea0dXdd9vqai=hGuQ8kuc9pgc9s8qqaq=dirpe0xb9q8qiLsFr0=vr0=vr0dc8meaabaqaciaacaGaaeqabaqabeGadaaakeaaiiGacqWF0oazdaWgaaWcbaGaemyAaKMaey4kaSIaeGymaedabeaakiabcIcaOiabdghaXjabcMcaPiabg2da9maaceaabaqbaeaabiGaaaqaamaaxababaGagiyBa0MaeiyyaeMaeiiEaGhaleaacuWGXbqCgaqbaaqabaGcdaGadaqaaiab=r7aKnaaBaaaleaaieGacqGFPbqAiiaacqqFRaWkieaacqaFXaqmaeqaaOGaeiikaGIafmyCaeNbauaacqGGPaqkcqWG0baDdaWgaaWcbaGafmyCaeNbauaacqWGXbqCaeqaaaGccaGL7bGaayzFaaGaeiilaWcabaGaeeyAaKMaeeOzayMaeeiiaaIae4xCaeNaeeiiaaIaeeyAaKMaee4CamNaeeiiaaIaeeyyaeMaeeiiaaIaeeizaqMaeeyzauMaeeiBaWMaeeyzauMaeeiDaqNaeeyzauMaeeiiaaIaee4CamNaeeiDaqNaeeyyaeMaeeiDaqNaeeyzauMaei4oaSdabaWaaCbeaeaacyGGTbqBcqGGHbqycqGG4baEaSqaaiqbdghaXzaafaaabeaakmaacmaabaGae8hTdq2aaSbaaSqaaiab+LgaPbqabaGccqGGOaakcuWGXbqCgaqbaiabcMcaPiabdsha0naaBaaaleaacuWGXbqCgaqbaiabdghaXbqabaaakiaawUhacaGL9baacqWGLbqzdaWgaaWcbaGaemyCaeNaeiilaWIaem4Cam3aaSbaaWqaaiabdMgaPjabgUcaRiabigdaXaqabaaaleqaaOGaeiilaWcabaGaee4Ba8MaeeiDaqNaeeiAaGMaeeyzauMaeeOCaiNaee4DaCNaeeyAaKMaee4CamNaeeyzauMaeeOla4caaaGaay5Eaaaaaa@9275@

Here, *q' *ranges over all states of the model, *t*_*q' q *_is the probability of the transition from state *q' *to state *q *and eq,si+1
 MathType@MTEF@5@5@+=feaafiart1ev1aaatCvAUfKttLearuWrP9MDH5MBPbIqV92AaeXatLxBI9gBaebbnrfifHhDYfgasaacH8akY=wiFfYdH8Gipec8Eeeu0xXdbba9frFj0=OqFfea0dXdd9vqai=hGuQ8kuc9pgc9s8qqaq=dirpe0xb9q8qiLsFr0=vr0=vr0dc8meaabaqaciaacaGaaeqabaqabeGadaaakeaacqWGLbqzdaWgaaWcbaGaemyCaeNaeiilaWIaem4Cam3aaSbaaWqaaiabdMgaPjabgUcaRiabigdaXaqabaaaleqaaaaa@354A@ is the probability of emitting nucleotide *s*_*i*+1 _out of state *q*. The Viterbi values can be computed by increasing *i *with the states sorted from left to right. By backtracking we can construct the most probable path Q* (see Figure 4) and thus the jumping alignment. This algorithm has a complexity of *O*(*nℓk*) in time and *O*(*nℓ*) in space where *n *is the length of the query sequence, *k *the number of subtypes in the alignment and *ℓ *the number of states in the jpHMM.

In the case of very long alignments this may require too much time and memory for current computer hardware. For example, genomes of the HIV-1 group M have a length of roughly 10, 000 nucleotides. Thus, given a multiple alignment of 14 (sub-)subtypes of such sequences and a query sequence of length ~10, 000 in a straightforward implementation we would need to calculate and store roughly 10, 000 · 10, 000 · 14 · 3 = 4 · 2 · 10^9 ^floating point numbers.

To accelerate the computation and to save memory we bound the number of considered states in each step *i *by using the beam-search algorithm [32,33]. The idea behind this algorithm is to exclude possible irrelevant paths in each step and to restrict the search space to 'promising' paths. If an alignment of an initial part is *much *worse than another alignment of that part then we do not try to extend that low quality alignment. This is achieved by computing and storing in each step *i *a modified Viterbi value δ′i(q)
 MathType@MTEF@5@5@+=feaafiart1ev1aaatCvAUfKttLearuWrP9MDH5MBPbIqV92AaeXatLxBI9gBaebbnrfifHhDYfgasaacH8akY=wiFfYdH8Gipec8Eeeu0xXdbba9frFj0=OqFfea0dXdd9vqai=hGuQ8kuc9pgc9s8qqaq=dirpe0xb9q8qiLsFr0=vr0=vr0dc8meaabaqaciaacaGaaeqabaqabeGadaaakeaaiiGacuWF0oazgaqbamaaBaaaleaacqWGPbqAaeqaaOGaeiikaGIaemyCaeNaeiykaKcaaa@3312@ ≤ *δ*_*i*_(*q*) only for a subset A
 MathType@MTEF@5@5@+=feaafiart1ev1aaatCvAUfKttLearuWrP9MDH5MBPbIqV92AaeXatLxBI9gBamrtHrhAL1wy0L2yHvtyaeHbnfgDOvwBHrxAJfwnaebbnrfifHhDYfgasaacH8akY=wiFfYdH8Gipec8Eeeu0xXdbba9frFj0=OqFfea0dXdd9vqai=hGuQ8kuc9pgc9s8qqaq=dirpe0xb9q8qiLsFr0=vr0=vr0dc8meaabaqaciaacaGaaeqabaWaaeGaeaaakeaaimaacqWFaeFqaaa@3821@_i _of the states, namely those states *q *whose modified Viterbi value is not much lower than the optimal local solution δi∗
 MathType@MTEF@5@5@+=feaafiart1ev1aaatCvAUfKttLearuWrP9MDH5MBPbIqV92AaeXatLxBI9gBaebbnrfifHhDYfgasaacH8akY=wiFfYdH8Gipec8Eeeu0xXdbba9frFj0=OqFfea0dXdd9vqai=hGuQ8kuc9pgc9s8qqaq=dirpe0xb9q8qiLsFr0=vr0=vr0dc8meaabaqaciaacaGaaeqabaqabeGadaaakeaaiiGacqWF0oazdaqhaaWcbaGaemyAaKgabaGaey4fIOcaaaaa@30CF@ = max_*q*_δ′i(q)
 MathType@MTEF@5@5@+=feaafiart1ev1aaatCvAUfKttLearuWrP9MDH5MBPbIqV92AaeXatLxBI9gBaebbnrfifHhDYfgasaacH8akY=wiFfYdH8Gipec8Eeeu0xXdbba9frFj0=OqFfea0dXdd9vqai=hGuQ8kuc9pgc9s8qqaq=dirpe0xb9q8qiLsFr0=vr0=vr0dc8meaabaqaciaacaGaaeqabaqabeGadaaakeaaiiGacuWF0oazgaqbamaaBaaaleaacqWGPbqAaeqaaOGaeiikaGIaemyCaeNaeiykaKcaaa@3312@ These states are called 'active' states and the set A
 MathType@MTEF@5@5@+=feaafiart1ev1aaatCvAUfKttLearuWrP9MDH5MBPbIqV92AaeXatLxBI9gBamrtHrhAL1wy0L2yHvtyaeHbnfgDOvwBHrxAJfwnaebbnrfifHhDYfgasaacH8akY=wiFfYdH8Gipec8Eeeu0xXdbba9frFj0=OqFfea0dXdd9vqai=hGuQ8kuc9pgc9s8qqaq=dirpe0xb9q8qiLsFr0=vr0=vr0dc8meaabaqaciaacaGaaeqabaWaaeGaeaaakeaaimaacqWFaeFqaaa@3821@_i _of active states of step *i *is determined by

Ai={q|δ′i(q)≥ℬδj∗},0<ℬ≪1.
 MathType@MTEF@5@5@+=feaafiart1ev1aaatCvAUfKttLearuWrP9MDH5MBPbIqV92AaeXatLxBI9gBaebbnrfifHhDYfgasaacH8akY=wiFfYdH8Gipec8Eeeu0xXdbba9frFj0=OqFfea0dXdd9vqai=hGuQ8kuc9pgc9s8qqaq=dirpe0xb9q8qiLsFr0=vr0=vr0dc8meaabaqaciaacaGaaeqabaqabeGadaaakeaafaqabeqacaaabaWenfgDOvwBHrxAJfwnHbqeg0uy0HwzTfgDPnwy1aaceaGae8haXh0aaSbaaSqaaiabdMgaPbqabaGccqGH9aqpcqGG7bWEcqWGXbqCcqGG8baFiiGacuGF0oazgaqbamaaBaaaleaacqWGPbqAaeqaaOGaeiikaGIaemyCaeNaeiykaKIaeyyzImRae8hlHiKae4hTdq2aa0baaSqaaiabdQgaQbqaaiabgEHiQaaakiabc2ha9jabcYcaSaqaaiabicdaWiabgYda8iab=XsicjablQMi9iabigdaXiabc6caUaaaaaa@5524@

The modified Viterbi value of the inactive states is set to 0, and does not need to be stored. In the next step *i *+ 1 of the recursion the modified Viterbi value δ′i+1(q)
 MathType@MTEF@5@5@+=feaafiart1ev1aaatCvAUfKttLearuWrP9MDH5MBPbIqV92AaeXatLxBI9gBaebbnrfifHhDYfgasaacH8akY=wiFfYdH8Gipec8Eeeu0xXdbba9frFj0=OqFfea0dXdd9vqai=hGuQ8kuc9pgc9s8qqaq=dirpe0xb9q8qiLsFr0=vr0=vr0dc8meaabaqaciaacaGaaeqabaqabeGadaaakeaaiiGacuWF0oazgaqbamaaBaaaleaacqWGPbqAcqGHRaWkcqaIXaqmaeqaaOGaeiikaGIaemyCaeNaeiykaKcaaa@34E4@ needs only be computed for states, which can be reached from a state in the subset of active states A
 MathType@MTEF@5@5@+=feaafiart1ev1aaatCvAUfKttLearuWrP9MDH5MBPbIqV92AaeXatLxBI9gBamrtHrhAL1wy0L2yHvtyaeHbnfgDOvwBHrxAJfwnaebbnrfifHhDYfgasaacH8akY=wiFfYdH8Gipec8Eeeu0xXdbba9frFj0=OqFfea0dXdd9vqai=hGuQ8kuc9pgc9s8qqaq=dirpe0xb9q8qiLsFr0=vr0=vr0dc8meaabaqaciaacaGaaeqabaWaaeGaeaaakeaaimaacqWFaeFqaaa@3821@_i _through a path with one emission. This speeds up the computation of the recursion.

**Figure 5:**
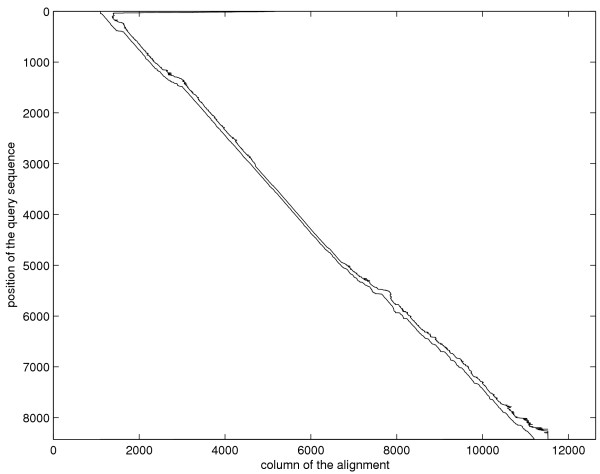
Reduction of active states for a set of HIV test sequences using the beam-search heuristics [32, 33]. In this example, we have 14 (sub-)subtypes each of which has three states per alignment column (match, delete and insert). Thus, a column corresponds to 14 × 3 = 42 states. The beam-search algorithm reduces the number of active states considerably; the figure indicates for each position in the query sequence those columns that contain active states. Thus, instead of considering the entire dynamic-programming matrix, our algorithm needs to consider only the small strip between the two lines. We used a beam width of ℬ
 MathType@MTEF@5@5@+=feaafiart1ev1aaatCvAUfKttLearuWrP9MDH5MBPbIqV92AaeXatLxBI9gBamrtHrhAL1wy0L2yHvtyaeHbnfgDOvwBHrxAJfwnaebbnrfifHhDYfgasaacH8akY=wiFfYdH8Gipec8Eeeu0xXdbba9frFj0=OqFfea0dXdd9vqai=hGuQ8kuc9pgc9s8qqaq=dirpe0xb9q8qiLsFr0=vr0=vr0dc8meaabaqaciaacaGaaeqabaWaaeGaeaaakeaaimaacqWFSeIqaaa@377E@ = 10^-20 ^and a jump probability of 10^-9^.

**Figure 6:**
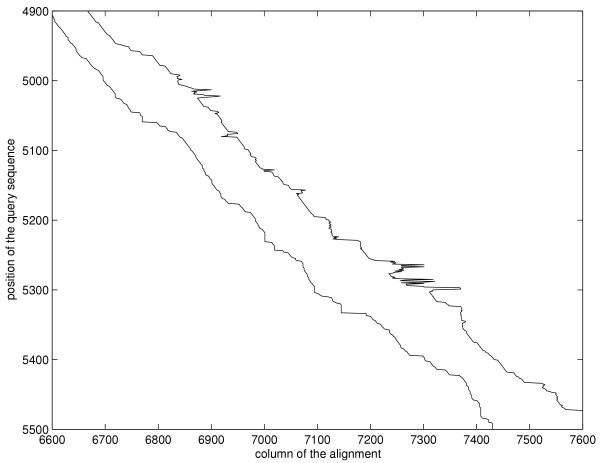
Detailed section of Figure 5.

In the tradeoff between memory efficiency and speed (large ℬ
 MathType@MTEF@5@5@+=feaafiart1ev1aaatCvAUfKttLearuWrP9MDH5MBPbIqV92AaeXatLxBI9gBamrtHrhAL1wy0L2yHvtyaeHbnfgDOvwBHrxAJfwnaebbnrfifHhDYfgasaacH8akY=wiFfYdH8Gipec8Eeeu0xXdbba9frFj0=OqFfea0dXdd9vqai=hGuQ8kuc9pgc9s8qqaq=dirpe0xb9q8qiLsFr0=vr0=vr0dc8meaabaqaciaacaGaaeqabaWaaeGaeaaakeaaimaacqWFSeIqaaa@377E@) against accuracy (small ℬ
 MathType@MTEF@5@5@+=feaafiart1ev1aaatCvAUfKttLearuWrP9MDH5MBPbIqV92AaeXatLxBI9gBamrtHrhAL1wy0L2yHvtyaeHbnfgDOvwBHrxAJfwnaebbnrfifHhDYfgasaacH8akY=wiFfYdH8Gipec8Eeeu0xXdbba9frFj0=OqFfea0dXdd9vqai=hGuQ8kuc9pgc9s8qqaq=dirpe0xb9q8qiLsFr0=vr0=vr0dc8meaabaqaciaacaGaaeqabaWaaeGaeaaakeaaimaacqWFSeIqaaa@377E@)) we chose a beam in the order that allows maximal accuracy within the limits of currenct PC hardware: ℬ
 MathType@MTEF@5@5@+=feaafiart1ev1aaatCvAUfKttLearuWrP9MDH5MBPbIqV92AaeXatLxBI9gBamrtHrhAL1wy0L2yHvtyaeHbnfgDOvwBHrxAJfwnaebbnrfifHhDYfgasaacH8akY=wiFfYdH8Gipec8Eeeu0xXdbba9frFj0=OqFfea0dXdd9vqai=hGuQ8kuc9pgc9s8qqaq=dirpe0xb9q8qiLsFr0=vr0=vr0dc8meaabaqaciaacaGaaeqabaWaaeGaeaaakeaaimaacqWFSeIqaaa@377E@ = 10^-20^. In Figure 5 and 6 we sketch the set of columns of activated states in the multiple alignment for an example with HIV sequences. Using this beam search heuristic very rarely affects the output of the computation but the time and memory savings are immense. The average number of active states per input sequence position in this example is 1,690 which compares to roughly 10,000 · 14 · 3 = 4 · 2 · 10^5 ^states if the beam search heuristic was not used. For the HIV-1 sequences that we tested, the average number of active states was between 1,620 (CRF 12, length = 8,760 nt) and 2,862 (CRF 11, length = 9,768 nt). The CPU time for those sequences using the beam search heuristics is 7.2 min (CRF 12) and 13.6 min (CRF 11) on a Linux PC with 3 GB RAM and 3.2 GHz. This includes model building as well as a search of one sequence against the model, but most of the CPU time was consumed by the second step.

### Test results

#### Results on HIV genomic sequences

To evaluate the accuracy of our jpHMM approach on HIV-1 sequences, we used two different types of test data including simulated as well as real-world sequence data.

First, we wanted to know to what extend our method is able to recognize subtypes in artificial sequences produced by the underlying probabilistic model itself. This test can be considered as a minimal check of our model. We sampled 800 random sequences according to the transition and emission probabilities of the HMM built for HIV-1 subtyping. Since the "jump probability" in our model is rather small, each of our 800 artificial sequences consisted of one subtype only without any recombinations. For each of these sequences our method correctly predicted the underlying single subtype, and no jumps between different subtypes were predicted. Moreover, for 752 of our 800 sequences, 100 % of the individual sequence positions were assigned to the correct subtype. In the remaining 48 sequences, the only differences between the sampled and predicted paths were in the lengths of short unclassified regions at the ends of the sequence. All in all, 99.99 % of the sequence positions in our test sequences were assigned to the correct subtype.

Further, we used *simulated *inter-subtype recombinant sequences with known breakpoints. Artificial recombinant sequences were created in the following way: for each simulated sequence, two real-world 'parent' sequences were taken from two different clades of HIV-1. For a fixed value *X*, these sequences were split at every *X*-th nucleotide, and a simulated recombinant sequence was composed of alternating segments of length *X *from these two parent sequences. Thus, for two parent sequences *P*1 and *P*2 and, for example, *X *= 1000, the first 1000 nucleotides of the artificial recombinant are from *P*1, nucleotides 1001 to 2000 are from *P*2, residues 2001 to 3000 are from *P*1 etc. In the present study, we used values of *X *= 500, 1000, 1500. This way, known breakpoints were introduced into both relatively conserved regions, and highly variable regions, and the performance of the jpHMM method could be assessed in both contexts. Here, we distinguish between recombinant sequences with parents from different *subtypes *which we call *inter-subtype *recombinants and recombinants with parents from the same subtype but different *sub-subtypes*, which we refer to as *inter sub-subtype *recombinants. Sub-subtypes are clearly distinguishable, established lineages that occur within the subtypes, but do not have the minimal genetic distances required to be considered an independent subtype. For historical reasons the B and D clades are called subtypes, but in fact the distances between these two clades correspond to a sub-subtype distance [15].

**Figure 7:**
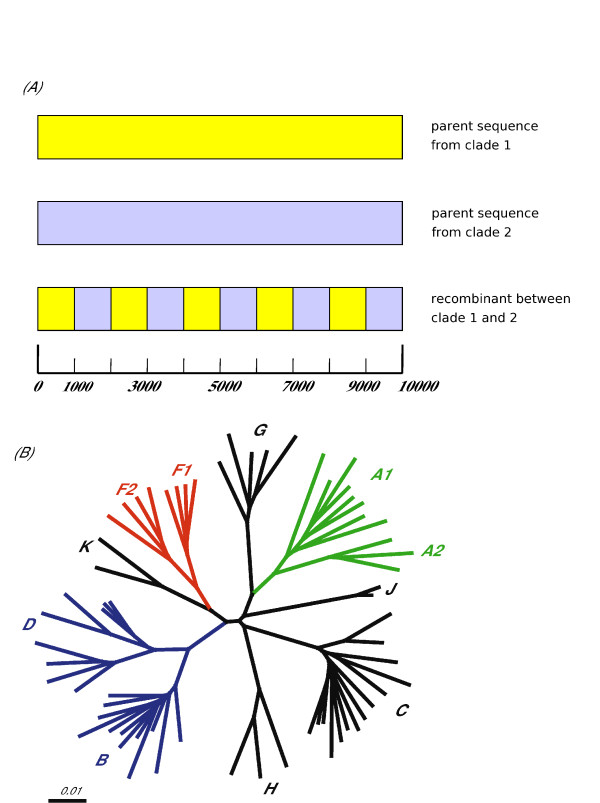
Creation of artificial recombinants with known breakpoints to test jpHMM and Simplot accuracy. (A) The artificial recombinant, constructed from two different clades, has the actual breakpoints at every X-th nucleotide for *X *= 500, 1000, 1500. Only the construction with *X *= 1000 is shown here. (B) The phylogenetic tree demonstrates the relations and relative distances between the clades used in the artificial recombinants' construction.

The sequences used in the inter-subtype recombinants have been created using parent sequences from the following subtypes (GenBank accession numbers of the parent sequences are in parentheses): A1 and C (A1: AF193275, C:AY463217); A1 and D (A1: AF193275, D:AF133821); A1 and G (A1: AF193275, G: AF450098); B and C (B: AF042101, C: AY463217); B and F1 (B: AF042101, F1:AY173958); B and 01 (B: AF042101, 01:AB032741). The sequences used in the inter sub-subtype artificial recombinants have been created from the following sub-subtypes and parents, respectively: A1 and A2 (A1: AF413987, A2:AF286240); A2 and A1 (A2:AF286241, A1:AF539405); B and D (B: AF538302, D:AJ320484); D and B (D: AJ488926, B:AY352275); F1 and F2 (F1:AY173957, F2:AF377956); F2 and F1 (F2: AY371158, F1:AY173958). We selected the above combinations of subtypes for the parent sequences, because they correspond to known real-world recombinants. From each subtype, we selected parent sequences for which breakpoints are assumed to be reliably annotated. Figure 7A illustrates the creation of these artificial recombinants, Figure 7B shows the evolutionary relations of the subtypes and inter sub-subtypes used for our simulated recombinants.

**Figure 8:**
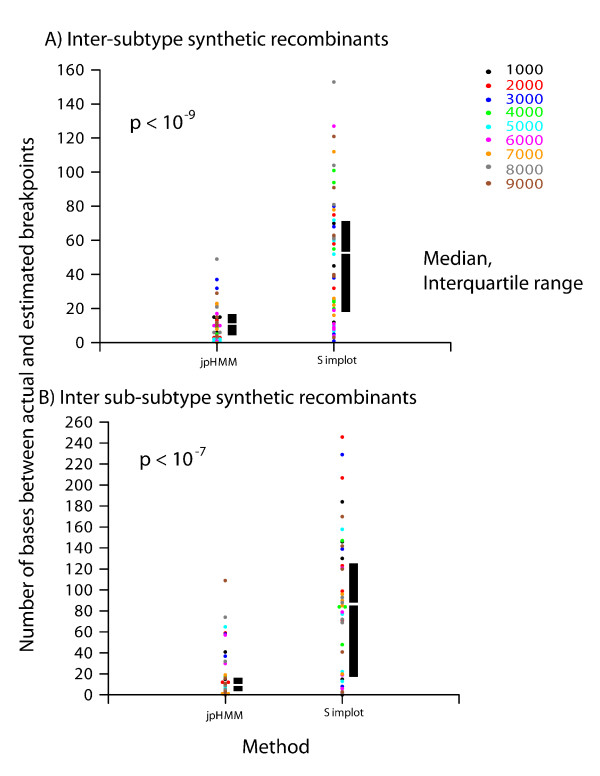
Evaluation of breakpoints predicted by jpHMM and Simplot. We measured the distance between the predicted breakpoints and the real breakpoints as described in the literature. For these distances, we calculated the median value and the interquartile range. A) Inter-subtype artificial recombinants: jpHMM's median is 10, with the interquartile range of 4–15; Simplot's median is 54, with the interquartile range of 19–72 and *p <*10^-9 ^in the Wilcoxon signed-rank test. B) Inter sub-subtype artificial recombinants: jpHMM's median values 9, interquartile ranges 3.5–19; Simplot's median values 84, interquartile ranges 19.5–122 and *p <*10^-7 ^in the Wilcoxon signed-rank test. In both plots (A and B), the *Y *-axis represents the number of bases that predicted breakpoints were away from the actual ones. The median values are shown here as the white bars, and the interquartile ranges are shown as the black bars. Numbers in color represent the actual breakpoint positions (every 1000-th nucleotide) in all synthetic recombinants. The duplicated data positions were spread apart in order to show every individual breakpoint here.

Based on these artificial recombinants, we evaluated the performance of our jpHMM tool and compared it with *Simplot *[12], a widely used HIV subtyping tool. We measured the distances between the predicted and the real breakpoints, and assessed the differences in prediction accuracy using non-parametric statistics, namely calculating the (*a*) the *median *value of the distances and (*b*) the *interquartile range*, and comparing distributions using the Wilcoxon signed-rank test implemented with R . As shown in Figure 8, our method consistently showed much better predictions of the artificial recombinant breakpoints than Simplot. In the inter-subtype sequences, jpHMM's median value for the distances between the predicted positions and the real breakpoints is 10, with an interquartile range from 4 to 15. By contrast, Simplot's median is 54, while its interquartile range is from 19 to 72. The difference between the predictions of jpHMM and Simplot is significant with *p <*10^-9 ^in the Wilcoxon signed-rank test.

The inter sub-subtype simulated sequences are more similar to each other, and so it becomes a more difficult problem to distinguish breakpoints. Here, jpHMM's median value for the distances between the predicted and the actual breakpoints is 9, the interquartile range is from 3.5 to 19, while Simplot's median value is 84 and its interquartile range is 19.5 to 122. The accuracy difference between jpHMM and Simplot's predictions are significant with *p <*10^-7 ^in the Wilcoxon signed-rank test. Finally, Figure 8 also shows there were no particular breakpoints that were consistently hard to define, rather whether or not a particular breakpoint (for example, position 1000) was accurately resolved depended on the particular combination of sequences; the artificial breakpoints we introduced were embedded in both conserved and variable regions of HIV. Introducing breakpoints at intervals of 500 and 1500 gave comparable results (data not shown) to the 1000 base intervals included in Figures 7 and 8. Finally, the breakpoint definition methods in Simplot uses a chi squared statistic for resolution [34].

We have tried to compare jphmm and Simplot chi square results to the suite of programs available in the RDP package [35], including RDP, GENECONV, MaxChi, Chimaera, Siscan. While Simplot and jphmm readily recognized the artificially generated breakpoints in our recombinants, shown in Figure 7, and could distinguish the parental subtypes, the other methods missed many of the breakpoints and often assigned incorrect subtype designations. The LARD [36] program appears not to be designed for recognition of multiple breakpoints. Bootscanning works well for correct identification of the subtypes of parental fragments in a recombinant genome, whether using the Simplot or RDP implementation, but does not attempt to optimally resolve breakpoints. Another algorithm to detect recombination events has been described in [37,38]. However, this method is limited to detect chimeric sequences that are recombinations of only *two *sequences with only one breakpoint. While the jumping alignment program JALI [8,9] can be adapted to run on DNA sequences, its computer memory requirements are far too high for applying it to the test data in our study. Thus we used the chi squared method [11,34] implemented in Simplot [12] for Figure 8, as it gave the best results among existing methods.

**Figure 9:**
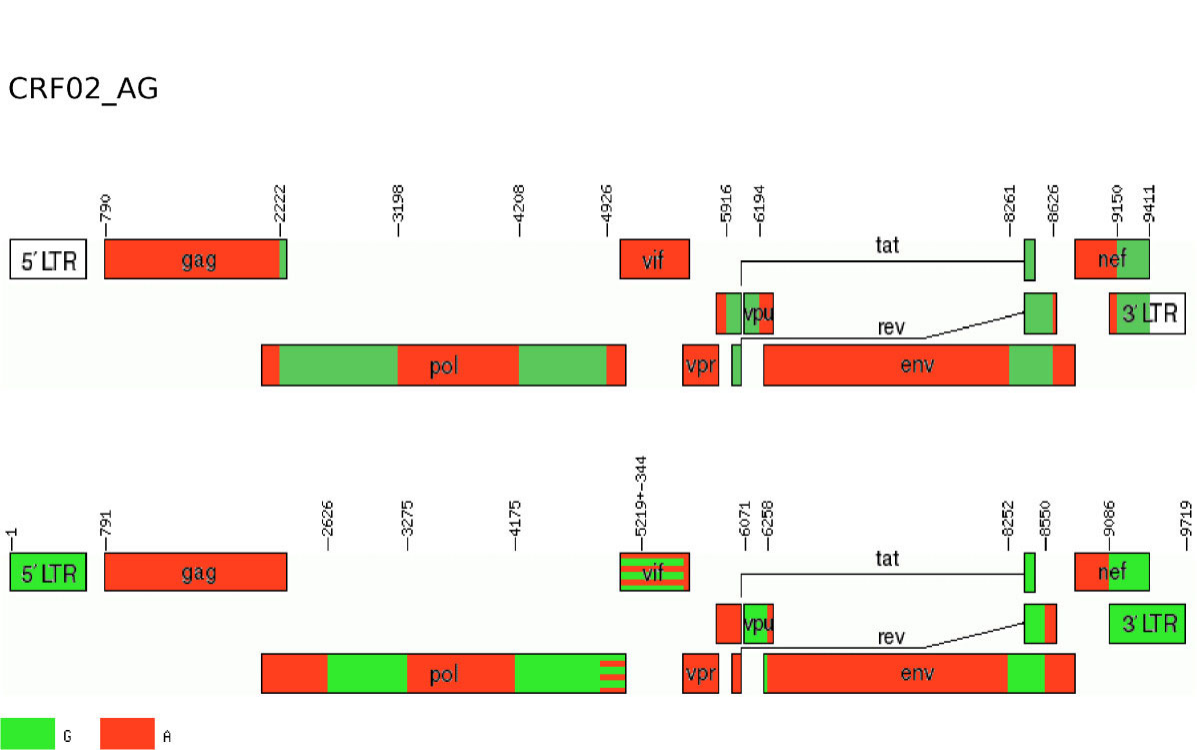
Comparison of genomic recombinations predicted by our jpHMM tool and reported in the literature. In all reference sequences from CRF02 to CRF08 and CRF10 to CRF14, 70% of the jpHMM's predictions were consistent with the published data. As an example, the figure shows the CRF02 recombinant that consists of subtypes A (shown in red) and G (shown in green). Above is the prediction by jpHMM, below the recombination as reported in the literature (see [42]).

In addition to simulated recombinants, we used real-world *circulating recombinant forms (CRFs) *for which the recombination breakpoints have been very carefully defined, and published in the literature. Here, we compared only our jpHMM predictions to the published data but not predictions by Simplot, since the published breakpoints already mainly rely on predictions by *Simplot *or similar methods. Thus, it would be redundant to compare CRFs to our own revised Simplot predictions. We tested reference sequences from 12 different CRFs, namely CRF02 to CRF08 and CRF10 to CRF14. These recombinants are known to be well annotated. Figure 9 shows the published genome map of CRF02 together with the subtypes as predicted by our jpHMM software. For the 12 CRFs that we analyzed, subtypes predicted by jpHMM roughly correspond to the previously published subtypes: for 70% of the CRFs with breakpoints, the breakpoints predicted by jpHMM are located in a distance of *<*150*nt *from the corresponding published breakpoints (with an average distance of 27*nt*). Discrepancies between the published and jpHMM predicted recombinant sequences were found in the H, J and K-containing CRFs.

#### Results for the hepatitis C virus

We analyzed the two recombinant strains of HCV that were available until mid-2005, the 1b/2k St Petersburg recombinants (AY587845, [22]) and an artificial 1a/2a recombinant (AF177037, [39]). In both cases, the jpHMM method accurately reconstructed the recombinant. jpHMM located the breakpoint for the 1b/2k St Petersburg recombinant between nucleotide 3186 and 3187 (in HCV-H77 numbering). The original authors manually pinpointed this breakpoint to the exact same nucleotide, based on a Simplot graphic and a sequence alignment.

The location of the breakpoint of the artificial 1a/2a recombinant was estimated to be at position 2759/2760 (HCV-H77 numbering), while the cross-over point in the actual artificial recombinant was reported to be between the fourth and fifth nucleotide of a mutated restriction site Nde 1 located at position 2761–66 of their reference sequence (the boundary of p7 and NS2). However, in the same reference sequence (AF177037) the only site (CATATG) was located at positions 2773–2778, which corresponds to 2762–2767 of HCV-H77. This would place the breakpoint at position 2765/2766, so in this case the prediction is off by 6 nucleotides.

In both recombinants, the genotype of the 5'UTR part of the sequence was misidentified, as 1a for the 1a/2a recombinant and as 2a for the 1b/2k recombinant. Thus, a spurious breakpoint was postulated for both sequences, at position 238/239 for the 1b/2k recombinant and at position 349/350 for the 1a/2a recombinant. This region of the HCV genome, around 350 nucleotides long, is known to be too highly conserved to contain a good phylogenetic signal, and often cannot even be used to phylogenetically distinguish different genotypes, let alone subtypes (CK, unpublished results), so it is not surprising that the jpHMM method is unable to make an accurate determination. As a consequence, for any automatic recombination detection method to work accurately, we expect that this region will have to be excluded a priori; and conversely, because this region does contain little phylogenetic information, detection of recombination will be almost impossible.

## Discussion and conclusions

We developed *jpHMM*, a novel probabilistic approach to compare DNA or protein sequences to a family of aligned sequences. In this study, we applied this tool to sequence subtyping and classification to enhance viral sequence quality control in the rapidly expanding HIV/HCV sequence databases. jpHMM combines the idea of a profile HMM with the jumping-alignment approach that has been previously proposed by Spang *et al*. [9]. For HIV and HCV genome sequences, we constructed profile HMMs for each subtype of the respective virus; these models were then connected by subtype transitions (jumps). These jumps make it possible to detect whether a query sequence is an inter-subtype recombinant by finding a best reference subtype match at each position along the entire query sequence. The results presented in this paper demonstrate jpHMM sensitively recognizes recombinants and gives more accurate breakpoint predictions than Simplot, a widely used subtyping tool in HIV-1 sequence analysis.

As every probabilistic model, jpHMM depends on a sufficiently large set of input data; our approach is therefore limited by the subtype background sets which are used as the model-building sequences. Our method performs best with large input data sets to inform the model, but it may fail to identify breakpoints if the input data set is too small. In the present study, for example, jpHMM failed on H, J, and K-containing CRFs. The difficulties with these sequences could be due to the following reasons: (1) jpHMM underestimates H, J, and K subtypes due to the fact that they have very rarely been sampled and sequenced, and so there are inadequate complete genome sequences from these three subtypes to develop a good model. (2) The current H, J, and K subtype reference sequences probably are not good representatives for these three subtypes, thus our jpHMM, as other subtyping tools, can be biased predicting these particular subtypes.

Thus, in its current form, while jpHMM provides clearly superior accuracy in terms of breakpoint definitions when large subtype data sets were available for input, to resolve rare subtypes it would be best to use this tool in conjunction with RIP [40] or Simplot for *de novo *HIV classification of unknown sequences. In this way, recombinant fragments from rare subtypes could be detected if present, and more accurate breakpoint definitions between common subtypes would be possible. In addition, jpHMM, like many other subtyping tools, fails on sequence classifications in the situation where a new or unknown sequence is discovered because there is no reference sequence available. We are currently developing another method to solve this problem.

In the present study, we applied jpHMM to viruses; we tested it on HIV/HCV sequences. The method, however, has been developed as a generally applicable tool, so its application should not be considered only in viral genomes. It could be successfully used to DNA and protein sequences from other organisms with individual subtype's sequences available, and be used as one important part in understanding the role of recombination in evolution and molecular epidemiology, and ultimately for integration into sequence quality control pipelines as a standard step in sequence analysis.

## Availability and requirements

The jpHMM program was written in C++ and the source code is available free of charge from the authors on request. We set up a user-friendly WWW interface for the program at [41] which is described [42]. The circulating recombinant forms of HIV are listed on a web page [43] of the HIV Sequence Database.

## Authors' contributions

AKS developed, implemented and tested the jpHMM algorithm, MZ and CK evaluated the program on HIV and HCV data, TL, BK and BM guided the project, MS conceived the jpHMM approach and supervised the program development. Each author wrote a part of the manuscript. All authors read and approved the final manuscript.
